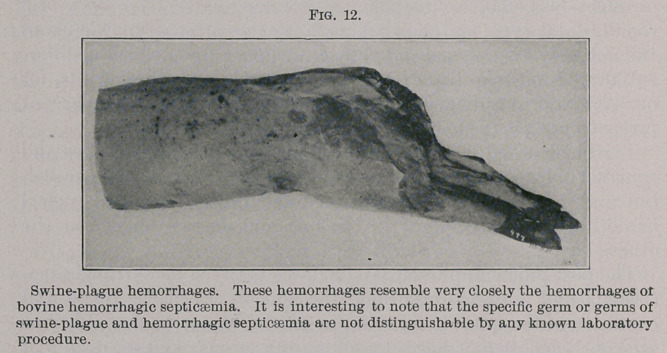# Hemorrhagic Septicæmia

**Published:** 1903-03

**Authors:** M. H. Reynolds

**Affiliations:** University of Minnesota, St. Anthony’s Park, Minn.


### HEMORRHAGIC SEPTICÆMIA.

By M. H. Reynolds, M.D., V.M.,
UNIVERSITY OF MINNESOTA, ST. ANTHONY'S PARK, MINN.

*Provisional Report on Bacteriological Examination of Hemorrhagic Septicæmia at State Experiment Station, St. Anthony's Park, June 9, 1902.*

(Concluded from page 95.)

*Suspected Outbreaks Not under the Personal Observation of the Writer.* Mr. W. L. Hoover, Faribault, called at my office on December 29th and said he had seventeen head of cattle coming two years of age and had lost four, the first case about December 1st, and the last one about December 27th. The first three died within a week, leaving quite an interval before the fourth one died, on December

Fig. 11.

Diaphragm showing typical hemorrhages.

27th. All of these animals died very suddenly. The owner noticed on skinning the animals hemorrhagic areas on the body surface, particularly on the neck, and stated that a similar condition may have been present in every case, but it was not noticed. He did not know whether similar areas had appeared upon the viscera or not.

Mr. E. G. Stark came to see me on December 29th concerning the loss of cattle in his neighborhood. He reported that Mr. Isaac

Carter had lost three cattle in about a week, out of a total of twelve head. This occurred just before Christmas. The first one died in about six hours after having been noticed sick. The two others also died very suddenly. He could give no information concerning the post-mortem conditions, but stated that the cattle had been fed on shocked corn and kept in the stable and yard.

Later I received a letter from Mr. Stark, dated January 28th, giving more definite information concerning the losses among Mr. Carter's cattle. He states that the first one died about December 10th. It had been found sick in the morning and died about 3.30 P.M. The second died about ten days later. On coming up a hill on its return the animal stumbled and fell dead, and, as he states, "it did not even kick after falling." This was a two-year-old steer. The third animal died about four days later. This one was taken sick at about 11 o'clock in the morning and lived until 4 o'clock the next morning, suffering very severely, at least so the owner supposed. This probably means that the animal did considerable struggling, and possibly groaned while down. The fourth animal was a young cow, and, as he expressed it, "she also died hard." About January 5th or 6th, two animals were found dead in the barn in the morning, and the next morning two more were found dead. None of these four last animals had been noticed sick. At the time of this outbreak Mr. Carter had sixteen head of cattle and lost eight. The owner informed Mr. Stark that those cases which had lived long enough to give an opportunity for observation had seemed very tender to the touch, particularly over the spinal column and near the base of the brain. The heads were drawn as far back as possible and the eyes "rolled up." The animals that died had been fed corn on the stalk.

One interesting bit of information in connection with this outbreak was to the effect that the owner had these eight animals drawn out just behind his stacks within a few yards of the barnyard after having removed the skin, and that no further cases appeared. One of the neighbors skinned most of these animals, for Mr. Carter had a nice lot of cattle, but his cattle received no infection.

Mr. Peter Nelson had lost eight and killed two out of a total of seventeen head. The first case appeared early in November. The deaths, with the exception of two, came very close together. These two died a week or so later. No careful examination post-mortem was made, and no further information was obtainable from Mr. Nelson.

Mr. Jens Sorensen, of Monticello, wrote me on December 7th concerning some disease among a neighbor's cattle. His letter was to the effect that a certain neighbor had lost eight cattle, and other neighbors had lost cattle from this disease. Some of these cattle, which the owners had supposed to be in perfect health, had dropped suddenly and died practically without struggling. Others have lived a few hours after being taken sick. The neighbors had noticed that those which lived for a few hours appeared very sensitive along the spinal column. This is very meagre information, of course.

Richard Anderson, Belle Plaine, had eleven head and lost four. The first one died about November 13th, the last one November 29th. None was sick at the time the information was received. The period of sickness was given as approximately three hours, but varied. The owner stated that the head was drawn backward after the animals went down; dark red areas were noticed under the skin, but no spots were seen on the internal organs. His cattle were confined to the yard and usually given dry feed, including shocked corn fodder. Mr. Anderson noticed also the peculiar grunting expiration, usual sensitiveness of the body surface under pressure, and that the animals were disinclined to walk around, being apparently sore. He described the typical condition of the intestines and rectal mucous membrane.

*Diagnosis.* It is very evident in view of the widely different types exhibited in different outbreaks, the very brief period of illness, and the similarity between this and certain other diseases, that a positive ante-mortem diagnosis is necessarily out of the question in many cases and uncertain in any case, except with the aid of previous autopsy and clear histories in previous cases of a given outbreak. The diagnosis must depend on the history, what little can be learned of the ante-mortem symptoms, and the results of examinations post-mortem. In all cases which came under the observation of the writer there were opportunities for such examinations, because all cases terminated fatally.

*Differential Diagnosis.* So far as facts occur to the writer at present, the differential diagnosis comes between anthrax, symptomatic anthrax, cornstalk disease, specific cerebro-spinal meningitis. There is no question in the writer's mind but that hemorrhagic septicæmia has in the past been very frequently diagnosed as other diseases, particularly as anthrax and symptomatic anthrax. The writer suspects that very many cases of so-called cornstalk disease have been nothing more or less than the disease now under discussion.

The differential diagnosis is perhaps shown as clearly in the preceding table as could be given in any other way.

The ante-mortem differential diagnosis, exclusive of laboratory findings, between hemorrhagic septicæmia and anthrax is to be made upon the appearance of the blood, the history of spread, the extent of spread, and temperature.

The ante-mortem differential diagnosis, exclusive of laboratory findings, between hemorrhagic septicæmia and anthrax must evidently be based upon the history of the cases, especially the ages of animals affected, temperature, local superficial lesions, and examination of blood taken from the tumor in case such lesion is present.

A discussion of ante-mortem diagnosis, exclusive of laboratory findings, between hemorrhagic septicæmia and specific cerebro-spinal meningitis of cattle is apparently not justified by existing reliable information concerning these two diseases. Our Minnesota outbreaks of infectious cerebro-spinal meningitis among cattle have been of a rapidly fatal type, and showing, so far as our present knowledge of the subject is concerned, no clinical evidence upon which a differential ante-mortem diagnosis could be made. In other words, for our ante-mortem diagnosis between these two diseases we are at present dependent almost wholly upon the laboratory.

It will be noted in studying the table that in their histories and general clinical evidences these two diseases run very closely parallel, but may be very easily distinguished at autopsy when the typical hemorrhagic lesions appear in the one and do not appear in the other.

The post-mortem differential diagnosis between hemorrhagic septicæmia and anthrax rests upon the appearance of the blood and condition of the spleen. In so far as general hemorrhagic conditions are concerned, hemorrhages involving the serous cavities, results of inoculation with the laboratory animals, and hemorrhages involving the heart or its membranes, and also in mortality, the diseases are very closely parallel indeed.

The post-mortem differential diagnosis between hemorrhagic septicæmia and symptomatic anthrax lies: in the appearance of multiple localized hemorrhagic areas in the former, but not in the latter, and emphysematous tumors involving the subcutaneous areolar and muscular tissues, especially of the upper portions of the limbs, which are frequently present in the latter but not in the former.

The post-mortem differential diagnosis between hemorrhagic septicæmia and cerebro-spinal meningitis, except in the cerebro-spinal type of the former, rests upon the involvement of the brain and cord, and their membranes, and upon the presence or absence of the typical hemorrhages elsewhere. Localized hemorrhages elsewhere in the one case or their absence in the other, should clear up the diagnosis as positively as would be possible without laboratory work. The differential diagnosis, so far as laboratory work is concerned, is apparently not particularly difficult, provided the work can be done under favorable conditions.

It is out of the question to discuss intelligently the differential diagnosis, either ante-mortem or post-mortem, between hemorrhagic septicæmia and cornstalk disease until we know something at least of what the latter is and have some definite information concerning it.

Treatment deserves no discussion, for, so far as our present information concerning the disease extends, it is a waste of time and medicine, although it is true that the two animals, Alzanka and Dell (University Farm outbreak) received full doses of nerve sedatives and lived much longer than other cases, but terminated in death just the same.

FIG. 12.

Swine-plague hemorrhages. These hemorrhages resemble very closely the hemorrhages of bovine hemorrhagic septicæmia. It is interesting to note that the specific germ or germs of swine-plague and hemorrhagic septicæmia are not distinguishable by any known laboratory procedure.

*General Conclusions.* For the present at least, we must consider the term "hemorrhagic septicæmia" as quite inclusive, a sort of generic name which must cover a multitude of widely varying types of disease, in all of which the specific micro-organism, B. bovisepticus is found, and so far as our present information is concerned, we are apparently justified in considering this germ as the specific cause of the widely varying types. It is also safe to assume that it is not by any means a new disease, the only new feature about it being probably its definite diagnosis by Dr. Wilson, of the Laboratory of the Minnesota State Board of Health. Very many outbreaks of this disease have unquestionably been diagnosed as cornstalk disease, black-leg, and anthrax. Those of us who have been so fortunate, or unfortunate perhaps, as to have had personal experience with the disease in Minnesota have had occasion to smile at the clearly described typical outbreaks of hemorrhagic septicæmia appearing in our veterinary journals under other names.

### A Comparative Study: Hemorrhagic Septicæmia, Anthrax, Symptomatic Anthrax, and Cerebro-spinal Meningitis.

| | Hemorrhagic septicæmia. | Anthrax. | Symptomatic anthrax. | Cerebro-spinal meningitis. |
|---|---|---|---|---|
| Aet. organism . | Bacillus bovisepticus. | Bacillus anthracis. | Bacillus chauvoei. | Diplococcus intercellularis; diplococcus pneumoniæ. |
| Infection . . . | Method unknown.[1] | Ingestion, inhalation, and inoculation. | Inoculation. | Uncertain, probably ingestion, possibly inhalation. |
| Extent of spread | Enzootic. | Epizootic. | Enzootic. | Enzootic. |
| How spread . . | Unknown. | Movement of any infected substance.[2] | Carcasses, stagnant water, feces, food, etc. | Uncertain, probably food stuffs in many cases. |
| Season favoring | Indifferent. | Wet spring, then dry, hot summer. | Most common in summer and fall. | No satisfactory information. |
| Susceptible animals | Cattle, sheep, horses, various wild animals.[3] | Nearly all domestic, many wild animals.[4] | Young cattle, sheep, goat.[5] | Cattle, horses, sheep, goat, and dog. |
| Laboratory animal inoculation | Generally fatal. | Generally fatal. | Rabbit and mouse resist. | D. intercellularis resisted by guinea-pig and rabbit. D. pneumoniæ generally fatal. |
| SYMPTOMS. | | | | |
| Onset . . . . | Sudden, except in rare chronic type. | Sudden. | Sudden. | Usually sudden. |
| Local lesion . . | Swellings, slight or absent. | Swellings may be present; absent in acute.[6] | Swellings usually present, and emphysematous. | Absent. |
| Urine . . . . | Sometimes blood stained. | Frequently blood stained or dark. | Sometimes blood stained or dark. | Normal appearance. |
| Feces . . . . | Often blood stained when alive several days. | Frequently blood stained or coated. | Sometimes blood stained. | |
| Temperature . | Frequently normal or subnormal.[7] | Very high early. | Very high early, may fall later. | Normal. |
| MORBID ANATOMY | | | | |
| Subcutaneous gas | Absent at death. | Absent at death, but decomposition rapid. | Present before death. | Absent. |
| Blood . . . . | Normal. | Dark, feeble coagulation; no reddening on exposure. | From general circulation normal; reddens on exposure.[8] | Normal. |
| Hemorrhages . | Usually present.[9] | Very general; nearly all organs subject. | Not general, but may occur in various tissues. | Reported present, but diagnosis questioned. |
| Serous cavities . | Walls frequently show small hemorrhages. | Reddish serum usually present | Reddish serum may be present.[10] | May contain reddish or lemon yellow serum. |
| Spleen . . . . | Normal, except superficial hemorrhages. | Enlarged, dark, soft. | Normal. | Normal. |
| Mortality . . . | 80–90 per cent. | 80–100 per cent. | 80–100 per cent. | 80–90 per cent. |

1 In some cases apparently by inoculation.
2 Including blood and discharges. Insects active agents of spread.
3 Possibly swine.
4 Pig but slightly susceptible.
5 Rare in cattle over two years old.
6 Not emphysematous.
7 Rising in some cases rapidly just before death.
8 From tumor dark frothy and fetid.
9 May vary greatly in size and intensity, but well defined.
10 Especially in the abdominal cavity.

## Figures and Tables

**Fig. 11. f1:**
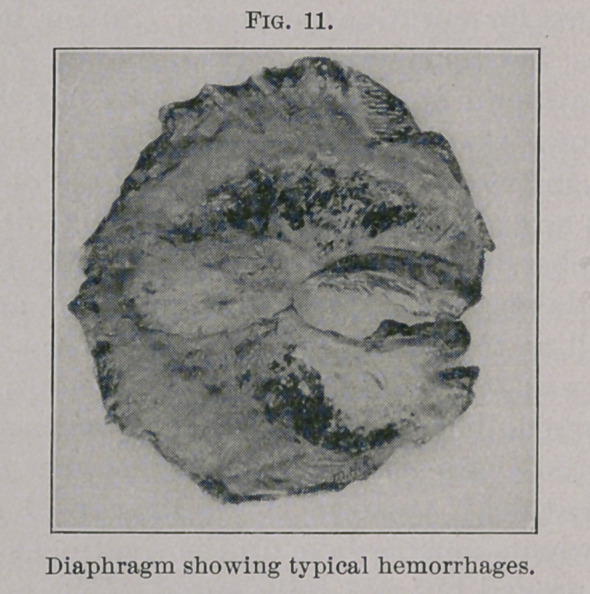


**Fig. 12. f2:**